# Identification of Blast Resistance QTLs Based on Two Advanced Backcross Populations in Rice

**DOI:** 10.1186/s12284-020-00392-6

**Published:** 2020-06-01

**Authors:** Haichao Jiang, Yutao Feng, Lei Qiu, Guanjun Gao, Qinglu Zhang, Yuqing He

**Affiliations:** National Key Laboratory of Crop Genetic Improvement and National Center of Plant Gene Research (Wuhan), Huazhong Agricultural University, Wuhan, 430070 China

**Keywords:** *Oryza sativa*, Rice blast, Mapping, Quantitative trait loci, Durable resistance

## Abstract

**Background:**

Rice blast is an economically important and mutable disease of rice. Using host resistance gene to breed resistant varieties has been proven to be the most effective and economical method to control rice blast and new resistance genes or quantitative trait loci (QTLs) are then needed.

**Results:**

In this study, we constructed two advanced backcross population to mapping blast resistance QTLs. CR071 and QingGuAi3 were as the donor parent to establish two BC_3_F_1_ and derived BC_3_F_2_ backcross population in the Jin23B background. By challenging the two populations with natural infection in 2011 and 2012, 16 and 13 blast resistance QTLs were identified in Jin23B/CR071 and Jin23B/QingGuAi3 population, respectively. Among Jin23B/CR071 population, 3 major and 13 minor QTLs have explained the phenotypic variation from 3.50% to 34.08% in 2 years. And, among Jin23B/QingGuAi3 population, 2 major and 11 minor QTLs have explained the phenotypic variation from 2.42% to 28.95% in 2 years.

**Conclusions:**

Sixteen and thirteen blast resistance QTLs were identified in Jin23B/CR071 and Jin23B/QingGuAi3 population, respectively. QTL effect analyses suggested that major and minor QTLs interaction is the genetic basis for durable blast resistance in rice variety CR071 and QingGuAi3.

## Introduction

Rice (*Oryza sativa*) is a staple food crop for more than 50% of the world’s population. Rice blast, caused by the fungal pathogen *Magnaporthe oryzae*, is one of the most serious diseases of rice in tropical and temperate areas of the world. Improving disease resistance in crops is crucial for stable food production. Nevertheless, using host resistance gene (R gene) to breed resistant varieties has been proven to be the most effective and economical method to control rice blast, and gene pyramiding is a promising method for providing broad-spectrum and durable resistance (Fukuoka et al. 2009; Liu et al. 2016; Tabien et al. 2002).

So far, over 100 blast resistant genes or quantitative trait loci (QTL) have been identified (Su et al. 2015; Vasudevan et al. 2016; Xiao et al. 2017; Zheng et al. 2016). Among them, 37 genes have been cloned (Wang et al. 2017; Wang et al. 2019; Zhao et al. 2018), and most of them belong to the nucleotide-binding site (NBS) leucine-rich repeat (LRR) gene family. Many of these R genes are clustered in the rice genome, especially on chromosomes 6, 11, and 12. Notably, at least 11 R genes have been identified, including *Pi2*, *Pi9*, *Pi22*, *Pi25*, *Pi26*, *Pi40*, *Pi42*, *Pigm*, *Piz*, *Pizt*, and *Pi50*, which are concentrated as gene clusters in the short-arm region near the centromere of chromosome 6. Of these, *Pi2*, *Pi9*, *Pi50*, *Pigm*, and *Pizt* have been cloned and have shown broad-spectrum resistance (Deng et al. 2017; Qu et al. 2006; Su et al. 2015; Zhou et al. 2006). It had been reported that at least 7 R genes were located in the long-arm of rice chromosome 11, including *Pik*, *Pi-kg(t)*, *Pikm*, *Pik-h*, *Pik-p*, *Pi54* and *Pi1* (Ashikawa et al. 2008; Hua et al. 2012; Pan et al. 1998; Sharma et al. 2010; Yuan et al. 2011; Zhai et al. 2011; Zhai et al. 2014). More than 20 R gene were located on rice chromosome 12, most of them were located near the centromere of chromosome 12, including *Pi-ta*, *Pi-ta*^*2*^, *Pi-ta*^*n*^, *Pi19*, *Pi20*, *Pi30*, *Pi31* and *Ptr* (Bryan et al. 2000; Hayashi et al. 1998; Imbe et al. 1997; Sallaud et al. 2003; Zhao et al. 2018).

Although the use of race-specific resistance genes is a major strategy for disease control, these genes are vulnerable to counter evolution of pathogens. Consequently, most varieties lose their resistance after a few years because of new *M. oryzae* races. Many studies indicated that the genetic control of blast resistance is complex and involves both major and minor resistance genes with complementary or additive effects, as well as environmental interactions (Bonman 1992; Li et al. 2007; Li et al. 2011; Wang et al. 1994; Wu et al. 2005). New resistance genes are then needed, thus continuing a cycle referred to as an evolutionary “arms race” between crops and pathogens (Jones and Dangl. 2006). Quantitative trait loci, which usually have smaller individual effects than R genes but confer broad-spectrum or non-race-specific resistance, can contribute to durable disease resistance (Kou and Wang. 2010). Thus, the discovery and use of novel QTLs and development of broad-spectrum resistant varieties are urgent goals in breeding for blast resistance in rice.

Most blast resistance genes confer complete and race-specific resistances that the highly variable fungus can overcome the R gene effects within 2 or 3 years after planting (Wang et al. 2017). The resistance conferred by R genes often do not support sustainable crop production. However, resistance controlled by partially effective resistance genes is often considered to be non race-specific and therefore durable. Durable resistance is the main goal of rice breeding, repeated observations generally suggest that cultivars carrying partial resistance maintain resistance for a long time, possibly because of decreased selection pressure upon the pathogen. CR071 and QingGuAi3 are *indica* rice (*Oryza sativa* L.) cultivar that has provided a high level of durable resistance to blast over past decades, and has been used as a donor of blast resistance in breeding in China. To understand the genetic mechanism of blast resistance in CR071 and QingGuAi3, two advanced backcross population BC_3_F_1_ and derived BC_3_F_2_ population from Jin23B/CR071 and Jin23B/QingGuAi3 were studied for blast response under conditions of natural infection. The objective was to find blast resistance loci in the donor parents and to explain the underlying mechanism of resistance. Such results should be useful for improving blast resistance in rice breeding.

## Materials and Methods

### Experimental Population

**Fig. 1:**
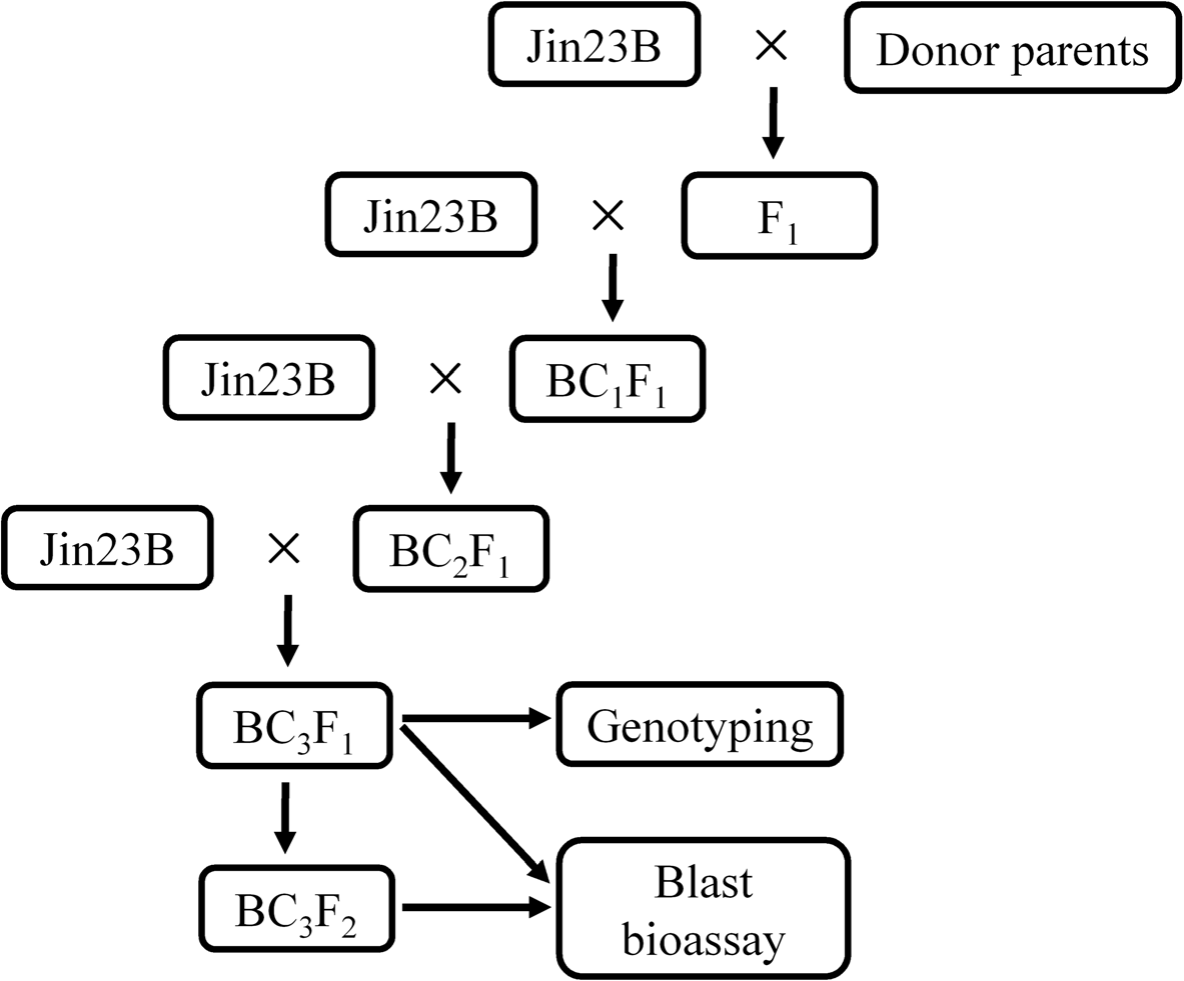
Strategy to develop mapping populations for blast resistance QTLs

The blast resistant *indica* cultivar CR071 and QingGuAi3 which provided by the Enshi Academy of Agricultural Science, Hubei, China, were used as the donor parent. CR071 and QingGuAi3 were blast resistance varieties which have a high and durable resistance to rice blast since 1983 (Wu et al. 2001). The blast susceptible *indica* cultivar Jin23B, the maintainer line for several elite hybrids in China, was used as the recurrent parent. Two backcross populations derived from the cross between Jin23B and CR071 and between Jin23B and QingGuAi3 were generated according to the plan outlined in Fig. 1. After the first cross, the F_1_ generations were backcross to Jin23B, then the BC_1_F_1_ seeds were sown in a blast nursery in Xianfeng County, Hubei province, in 2010. Resistance plants identified by their leaf blast reactions were further backcrossed with the recurrent parent Jin23B, and BC_2_F_1_ plants were obtained. The BC_2_F_1_ seeds were sown in Hainan province in 2011 and were randomly crossed with Jin23B (Fig. 1), two backcross population contained 239 and 237 plants, respectively were obtained. The BC_3_F_1_ plants and parents were planted during the normal rice growing seasons (from mid-May to early October) at the experimental field of Xianfeng for phenotypic measurement in 2011 and selfed to produce BC_3_F_2_ families. In 2012, the BC_3_F_2_ families, each containing 12 plants, were grown in Xianfeng County for blast phenotyping. Selected lines in the BC_3_F_1_ population were backcross to Jin23B to obtain BC_4_F_1_, and the self-cross seed of these BC_4_F_1_ plants were used to develop BC_4_F_2_ segregating population of each QTL. The BC_4_F_2_ segregating populations of *qBR3–3* and *qBR6* from CR071 and *qBR6* and *qBR7–1* from QingGuAi3 were planted in 2013 in Xianfeng. Varieties BL6 and CO39 were used as resistant and susceptible control.

### Trait Evaluations

All plants of the BC_3_F_1_ population were scored for leaf blast response at tillering and heading stages and were recorded for neck blast at maturation stages in 2011; the three traits were named 11RT (leaf blast response of plants at tillering, 2011), 11RH (leaf blast response of the plants at heading, 2011) and 11RN (neck blast of the plants at maturation, 2011). The BC_3_F_2_ populations were scored on an individual plant basis for leaf blast at tillering heading and for neck blast at the maturation stage, 2012, and results were named 12RT (leaf blast response of plants at tillering, 2012), 12RH (leaf blast response of plants at heading, 2012) and 12RN (neck blast of the plants at maturation, 2012). For the BC_3_F_2_ families, 24 plants of each family were planted in two row, and the middle 20 plants were scored for blast response. The mean of each family was used as raw data for QTL analysis. Lines developing asynchronously at the normal tillering or heading stages were excluded so that all data would reflect the same developmental stages of the plants. The BC_4_F_2_ segregation population were scored for leaf blast response at tillering stage in 2013. The most seriously diseased leaf of the top two or three new leaves was scored for each plant at each stage using the rating scale of Bonman et al. (1986), where 0 = no evidence of infection; 1 = brown specks smaller than 0.5 mm in diameter, no sporulation; 2 = brown specks about 0.5–1.0 mm in diameter, no sporulation; 3 = roundish to elliptical lesions, 1–3 mm in diameter, grey centre surrounded by brown margins, lesions capable of sporulation; 4 = typical spindle-shaped blast lesions capable of sporulation,3 mm or longer; 5 = lesions as in 4 but about half of one to two leaf blades killed by coalescence of lesions. Reaction types 0, 1, 2 and 3 were considered resistant, and 4 and 5 as susceptible (Das et al. 2012). Neck blast severity was recorded as a percentage of infection on the neck of rice panicle at physiological maturity stage. The number of panicles showing symptoms of neck blast was expressed as percent infection. Reaction types 0–25% were considered resistant, and 26%–100% as susceptible (Gao et al. 2008; IRRI 2002). To induce infection by the pathogen, diseased straw collected during the previous year was evenly dispersed in each plot and the highly susceptible variety; CO39, was planted on both sides of each row and around the experimental population. Field management essentially followed normal agricultural practices with the exception of no use of bactericides.

### DNA Markers

Among 1032 simple sequence repeat (SSR) markers, 182 were polymorphic between Jin23B and CR071, and 161 were polymorphic between Jin23B and QingGuAi3, the level of polymorphism were 17.63% and 15.60%, respectively. A total of 145 and 113 polymorphic markers covering the whole rice genome were used to develop the genetic linkage map of Jin23B/CR071 and Jin23B/QingGuAi3 population. The RM marker series were searched in the available rice genomic database (http://www.gramene.org). Insertion/deletion markers were designed based on the references maps of Nipponbare and 9311. Genomic DNA was isolated from leaf tissues using the CTAB method. The SSR assay was performed with 4% urea polyacrylamide gels migration and silver staining as reported by Panaud et al. (1996).

### Genetic Map Construction and QTL Analysis

A genetic linkage map was constructed using the Kosambi mapping function of MapMaker/Exp3.0 program (Lincoln et al. 1992). QTL analysis was performed by composite interval mapping (CIM) method using Windows QTL Cartographer 2.5 software (Wang et al. 2007) with a logarithm of odds (LOD) threshold of 2.5. Genotypes of the BC_3_F_1_ population were determined using SSR markers. The resistance score and genotype of each plant in the BC_3_F_1_ population were used for QTL analysis. For QTL detection of the BC_3_F_2_ population, the mean of each BC_3_F_2_ family was used as the row value and the genotypes of the BC_3_F_1_ plants were used as the genotypes of the BC_3_F_2_ families. Correlation analysis between six observation times in 2011 and in 2012 were examined by the Pearson correlation coefficient test. The resistance score of each plant in the BC_3_F_1_ population were used for correlation analysis. The mean of each BC_3_F_2_ family were used as the row value for correlation analysis. Data analyses were performed using Microsoft Excel 2003 or SPSS 17.0.

## Results

### SSR Assay of Two Backcross Population

Two backcross populations derived from the cross between Jin23B and CR071 and between Jin23B and QingGuAi3 contained 239 and 237 plants, respectively. Genomic DNA was isolated from young leaves of seeding from each plant using the CTAB method. A total of 145 polymorphic SSR markers covering the whole rice genome between Jin23B and CR071, and 113 polymorphic SSR markers between Jin23B and QingGuAi3 were used to detect the genotype of each plant. Theoretically, 87.5% of the markers are Jin23B homozygous genotype of each individual plant in BC_3_F_1_ population. In the Jin23B/CR071 background population, the marker ratio of Jin23B homozygous genotype of each plant were from 52.41% to 99.31%, most plants with a marker ratio in 90–100% (Fig. S1). In the Jin23B/QingGuAi3 background population, the marker ratio of Jin23B homozygous genotype of each plant were from 51.33% to 94.69%, most plants with a marker ratio in 80–90% (Fig. S1).

### Measurements and Relationship of the Traits

The receptor parent Jin23B is an *indica* variety with susceptible performance to rice blast, and the donor parents CR071 and QingGuAi3 with resistance performance to rice blast. The leaf blast resistance score of Jin23B were 4.17 and 4.11 at tillering stage, and 4.22 and 4.19 at heading stage in 2011 and 2012 respectively (Table 1). The neck blast resistance of Jin23B were 91.7% and 93.7% in 2011 and 2012 respectively (Table 1). The leaf blast resistance score of CR071 and QingGuAi3 were 0.83 and 1.06 at tillering stage in 2011, and 0.97 and 1.11 at tillering stage in 2012 (Table 1). The leaf blast resistance score of CR071 and QingGuAi3 were 1.00 and 1.14 at heading stage in 2011, and 1.00 and 1.19 at heading stage in 2012 (Table 1). For neck blast resistance, CR071 and QingGuAi3 were 3.33% and 6.67% in 2011, and 4.67% and 8.33% in 2012 (Table 1). The resistance score between CR071 and Jin23B, QingGuAi3 and Jin23B were significant different at the corresponding measurement stages (Table 1). The resistance control BL6 and susceptible control CO39 had leaf blast scores of 1.20 and 4.60 at tillering stage and 1.30 and 5.00 at heading stage in 2011. And those for neck blast of BL6 and CO39 at maturation stage were 10.11% and 96.53% in 2011, and 12.10% and 100% in 2012.

**Table 1:** Statistical description of the parents and the backcross population in 2 years

Trait	Year	Parent			Jin23B/CR071 backcross population		Jin23B/QingGuAi3 backcross population	
		Jin23B	CR071	QingGuAi3	Mean ± SD	Range	Mean ± SD	Range
RT	2011	4.17 ± 0.38	0.83 ± 0.38	1.06 ± 0.47	2.59 ± 1.12	0–5	2.30 ± 1.16	0–5
	2012	4.11 ± 0.32	0.97 ± 0.16	1.11 ± 0.40	2.58 ± 1.14	0.17–4.5	2.05 ± 1.05	0–4.75
RH	2011	4.22 ± 0.42	1.00 ± 0.23	1.14 ± 0.35	2.48 ± 1.25	0–5	2.37 ± 1.30	0–5
	2012	4.19 ± 0.40	1.00 ± 0.33	1.19 ± 0.40	2.59 ± 1.16	0–4.5	2.42 ± 1.08	0–4.58
RN	2011	91.7% ± 2.08%	3.33% ± 1.15	6.67% ± 0.58%	62.7% ± 40.7%	0–100%	71.3% ± 36.91%	0–100%
	2012	93.7% ± 2.31%	4.67% ± 1.52	8.33% ± 0.58%	54.8% ± 33.6%	0–100%	78.58 ± 32.23%	0–100%

Note: *RT* leaf blast resistance at tillering stage, *RH* leaf blast resistance at heading stage, *RN* neck blast resistance at maturation stage

**Fig. 2:**
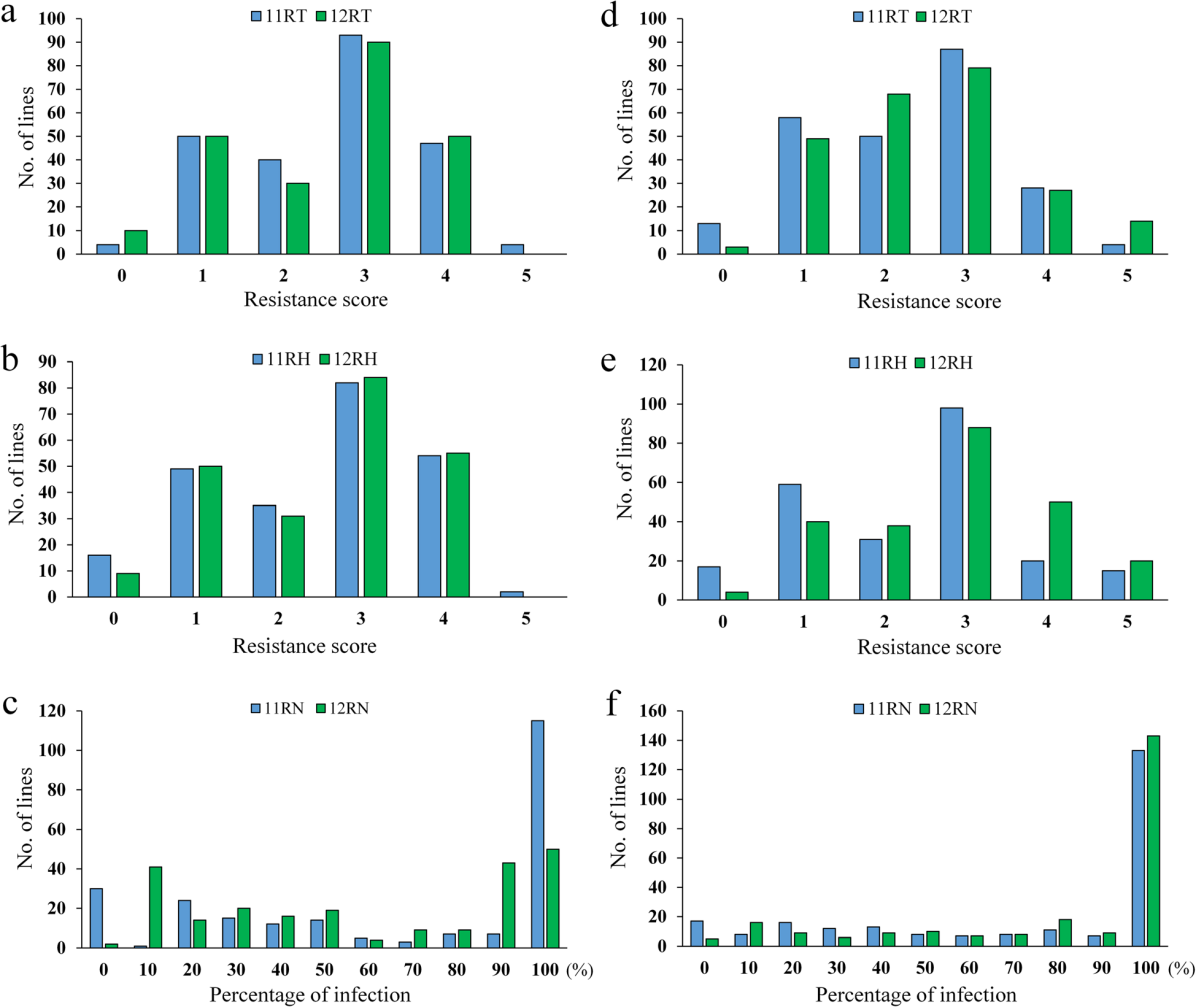
Frequency distribution of blast resistance of the Jin23B/CR071 (**a**, **b**, **c**) and Jin23B/QingGuAi3 (**d**, **e f**) population in 2011 and 2012. 11RT and 12RT, leaf blast resistance at tillering stage in 2011 and 2012. 11RH and 12RH, leaf blast resistance at heading stage in 2011 and 2012. 11RN and 12RN, neck blast resistance at maturation stage in 2011 and 2012. Blue pillar and green pillar indicate the frequency of BC_3_F_1_ and BC_3_F_2_ population, respectively

The distributions of lesion scores as measures of blast response at tillering and heading stages for leaf blast and maturation stage for neck blast for the BC_3_F_1_ population in 2011 and BC_3_F_2_ population in 2012 are shown in Fig. 2. There was transgressive segregation in both directions for all traits. The Pearson correlation coefficients showed significant correlation (*p* < 0.01) between all six traits in both years (Table S1; Table S2). Blast resistance of the BC_3_F_1_ population at the tillering and heading stages in 2011 had a remarkable positive relationship with blast resistance of the BC_3_F_2_ population at the heading and maturation stages in 2012 (*p* < 0.01). Resistance during different stages also exhibited significant relationships (*p* < 0.01). Leaf blast at the tillering and heading stages and neck blast at the maturation stage were significantly correlated with each other, so we were able to predict the neck blast response level according to the leaf blast response at tillering under natural condition.

### QTL Mapping for Blast Resistance in Jin23B/CR071 Population

**Fig. 3:**
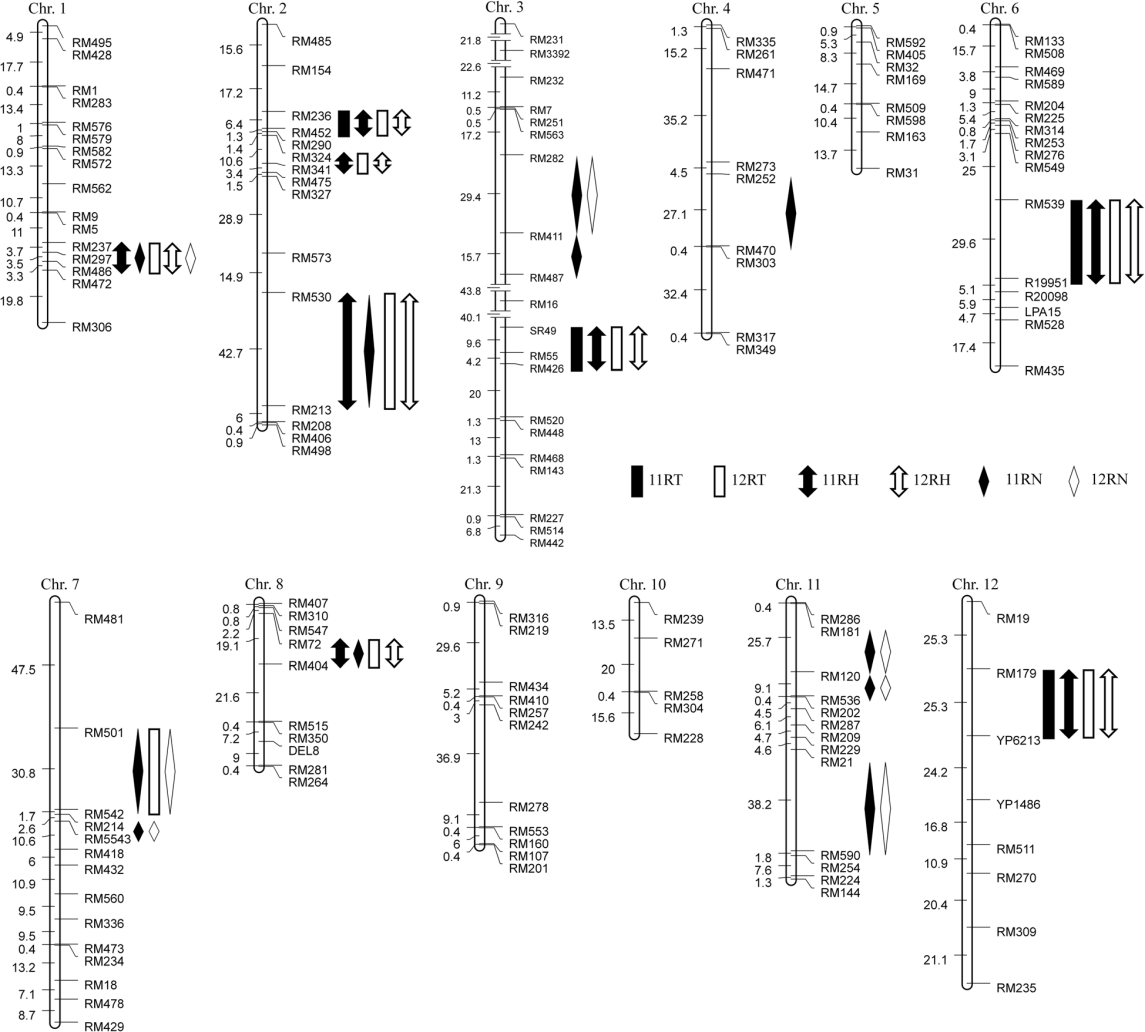
Distribution of QTLs for blast resistance in the Jin23B/CR071 population on the genetic linkage map. 11RT and 12RT, leaf blast resistance at tillering stage in 2011 and 2012. 11RH and 12RH, leaf blast resistance at heading stage in 2011 and 2012. 11RN and 12RN, neck blast resistance at maturation stage in 2011 and 2012

A total of 16 QTLs for blast resistance were identified on chromosomes 1, 2, 3, 4, 6, 7, 8, 11 and 12 in the Jin23B/CR071 population in 2 years (Table 2; Fig. 3). The phenotypic variance explained by each QTL ranged from 3.50% to 34.08%.

**Table 2:** QTL mapping results from Jin23B/CR071 background population in 2 years

Trait	Chr	QTL	Position	LOD	Add	*R*^*2*^ (%)	Trait	Chr	QTL	Position	LOD	Add	*R*^*2*^ (%)
11RT							12RT	1	*qBR1*^a^	RM237-RM486	7.39	-0.73^a^	6.09
	2	*qBR2–1*	RM236-RM452	5.32	1.42	6.64		2	*qBR2–1*	RM236-RM452	6.17	1.63	8.17
								2	*qBR2–2*	RM324-RM341	4.73	1.20	5.77
								2	*qBR2–3*	RM530-RM213	4.13	0.92	9.15
	3	*qBR3–3*	SR49-RM426	20.15	1.58	25.42		3	*qBR3–3*	SR49-RM426	30.08	1.81	34.08
	6	*qBR6*	RM539-R19951	4.05	0.92	8.89		6	*qBR6*	RM539-R19951	10.02	1.92	20.40
								7	*qBR7–1*	RM501-RM542	3.47	1.51	10.09
								8	*qBR8*	RM72-RM404	4.04	1.31	6.29
	12	*qBR12*	RM179-YP6213	2.80	0.57	4.25		12	*qBR12*	RM179-YP6213	3.57	0.58	4.48
11RH	1	*qBR1*^a^	RM237-RM486	9.34	−0.93	7.99	12RH	1	*qBR1*^a^	RM237-RM486	8.35	−0.80	7.25
	2	*qBR2–1*	RM236-RM452	3.90	1.41	4.51		2	*qBR2–1*	RM236-RM452	5.75	1.41	6.38
	2	*qBR2–2*	RM324-RM341	4.03	1.12	4.89		2	*qBR2–2*	RM324-RM341	3.91	1.13	5.21
	2	*qBR2–3*	RM530-RM213	4.51	0.96	8.68		2	*qBR2–3*	RM530-RM213	3.23	0.84	7.47
	3	*qBR3–3*	SR49-RM426	23.67	1.76	25.83		3	*qBR3–3*	SR49-RM426	28.23	1.78	31.33
	6	*qBR6*	RM539-R19951	8.94	0.85	7.79		6	*qBR6*	RM539-R19951	7.80	0.95	10.40
	8	*qBR8*	RM72-RM404	6.78	1.71	7.00		8	*qBR8*	RM72-RM404	4.20	1.30	4.83
	12	*qBR12*	RM179-YP6213	3.28	0.59	3.90		12	*qBR12*	RM179-YP6213	2.93	0.53	3.50
11RN	1	*qBR1*^a^	RM237-RM486	10.70	−0.36	8.12	12RN	1	*qBR1*^a^	RM237-RM486	10.55	−0.35	9.48
	2	*qBR2–3*	RM530-RM213	3.61	0.28	7.10							
	3	*qBR3–1*	RM282-RM411	4.74	0.31	7.19		3	*qBR3–1*	RM282-RM411	7.50	0.23	7.02
	3	*qBR3–2*	RM411-RM487	4.12	0.23	3.91							
	4	*qBR4*	RM252-RM470	3.04	0.22	4.16							
	7	*qBR7–1*	RM501-RM542	28.26	0.54	28.48		7	*qBR7–1*	RM501-RM542	20.27	0.36	18.94
	7	*qBR7–2*	RM214-M5543	25.26	0.48	22.00		7	*qBR7–2*	RM214-M5543	17.44	0.37	18.94
	8	*qBR8*	RM72-RM404	3.33	0.37	4.06							
	11	*qBR11–1*	RM181-RM120	27.25	0.58	28.16		11	*qBR11–1*	RM181-RM120	21.57	0.47	26.19
	11	*qBR11–2*	RM120-RM536	24.09	0.44	20.26		11	*qBR11–2*	RM120-RM536	18.69	0.34	17.93
	11	*qBR11–3*	RM21-RM590	8.02	0.53	17.48		11	*qBR11–3*	RM21-RM590	5.07	0.41	15.94

Note: *Chr* Chromosome, *LOD* logarithm of odds, *Add* the additive effect of each QTL, *R*^*2*^ Goodness of fit, represent the phenotypic variance explained by each QTL. ^a^, the resistance effect of QTL come from Jin23B. 11RT and 12RT, leaf blast resistance at tillering stage in 2011 and 2012. 11RH and 12RH, leaf blast resistance at heading stage in 2011 and 2012. 11RN and 12RN, neck blast resistance at maturation stage in 2011 and 2012

For leaf blast resistance at tillering stage, nine QTLs were detected on chromosome 1, 2, 3, 6, 7, 8 and 12 (Table 2; Fig. 3). Among them, four QTLs, *qBR2–1*, *qBR3–3*, *qBR6* and *qBR12* were detected in both year, five QTLs, *qBR1*, *qBR2–2*, *qBR2–3*, *qBR7–1* and *qBR8* were detected only in 2012 (Table 2; Fig. 3). The QTLs flanked by SR49 and RM426 on chromosome 3, *qBR3–3*, was detected in both year and explained 25.42% of the phenotypic variation in 2011 and 34.08% of the phenotypic variation in 2012. A QTLs, *qBR6*, located between RM539 and R19951 on chromosome 6, was also detected in 2 years and explained 8.89% and 20.40% of the phenotypic variation, respectively. Two QTLs, *qBR2–1* and *qBR12*, were located flanked by RM236-RM451 on chromosome 2, and RM179-YP6213 on chromosome 12, respectively. *qBR2–1* was detected in both years and accounted for 6.64% and 8.17% of the phenotypic variation, respectively. Whereas *qBR12* was detected in both year and accounted for 4.25% and 4.48% of the phenotypic variation.

For leaf blast resistance at heading stage, eight QTLs were detected on chromosome 1, 2, 3, 6, 8 and 12 (Table 2; Fig. 3), and all the QTLs were detected in both year. These QTLs explained the phenotypic variance ranged from 3.90% to 25.83% in 2011, and from 3.50% to 31.33% in 2012. Among them, the QTL, *qBR3–3*, flanked by SR49 and RM426 on chromosome 3, have the largest effect which explained 25.83% of the phenotypic variation in 2011 and 31.33% of the phenotypic variation in 2012.

For neck blast resistance at maturation stage, eleven QTLs were detected on chromosome 1, 2, 3, 4, 7, 8 and 11 (Table 2; Fig. 3). Among them, seven QTLs, *qBR1*, *qBR3–1*, *qBR7–1*, *qBR7–2*, *qBR11–1*, *qBR11–2* and *qBR11–3* were detected in both year, four QTLs, *qBR2–3*, *qBR3–2*, *qBR4* and *qBR8* were detected only in 2011 (Table 2; Fig. 3). These QTLs explained the phenotypic variance ranged from 3.91% to 28.48% in 2011, and from 7.02% to 26.19% in 2012. A QTL, *qBR7–1*, located between RM501 and RM542 on chromosome 7, explained 28.48% of the phenotypic variation, which have the largest effect in 2011. Whereas the QTL *qBR11–1*, located between RM181 and RM120 on chromosome 11, accounted for 26.19% of the phenotypic variation, which have the largest effect in 2012.

Among the sixteen QTLs in the Jin23B/CR071 population, the gene effect of fifteen QTLs come from donor parent CR071, in contrast, the gene effect of *qBR1* come from Jin23B. In the population, some plants have a transgressive segregation. Through analysis, we found that the QTL *qBR1* has a negative effect. So the gene effect of *qBR1* is from Jin23B, not CR071. The QTLs, *qBR1*, flanked by RM237 and RM486 on chromosome 1, was detected in both year for leaf blast and neck blast resistance and explained phenotypic variations of 6.09% in 12RT, 7.99% in 11RH, 7.25% in 12RH, 8.12% in 11RN and 9.48% in 12RN, respectively (Table 2). Two QTLs *qBR2–3* and *qBR8* were detected in both year for leaf blast and neck blast resistance; five QTLs *qBR2–1*, *qBR2–2*, *qBR3–3*, *qBR6* and *qBR12* were detected in both year for leaf blast at tillering and heading stages; eight QTLs *qBR3–1*, *qBR3–2*, *qBR4*, *qBR7–1*, *qBR7–2*, *qBR11–1*, *qBR11–2* and *qBR11–3* were detected only for neck blast in maturation stage.

### QTL Mapping for Blast Resistance in Jin23B/QingGuAi3 Population

**Fig. 4:**
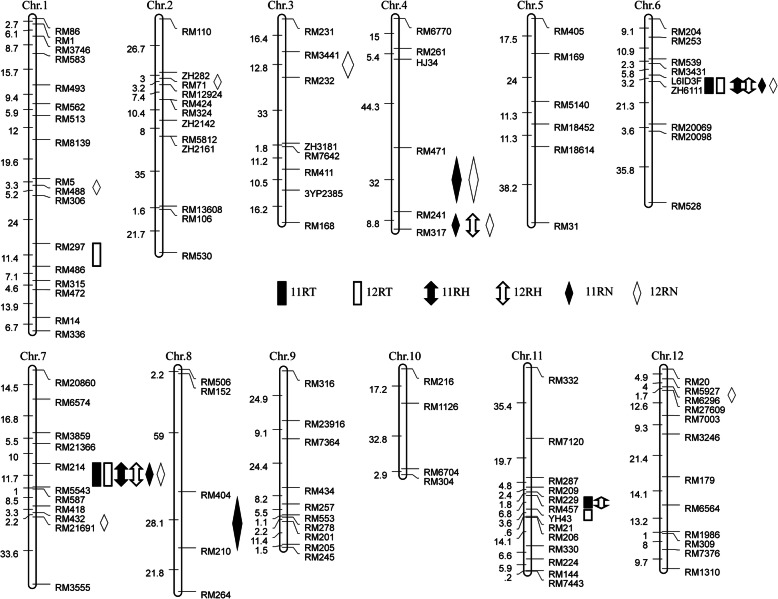
Distribution of QTLs for blast resistance in the Jin23B/QingGuAi3 population on the genetic linkage map. 11RT and 12RT, leaf blast resistance at tillering stage in 2011 and 2012. 11RH and 12RH, leaf blast resistance at heading stage in 2011 and 2012. 11RN and 12RN, neck blast resistance at maturation stage in 2011 and 2012

A total of 13 QTLs for blast resistance were identified on chromosomes 1, 2, 3, 4, 6, 7, 8, 11 and 12 in the Jin23B/QingGuAi3 population in 2 years (Table 3; Fig. 4). The phenotypic variance explained by each QTL ranged from 2.42% to 28.95%.

**Table 3:** QTL mapping results from Jin23B/QingGuAi3 background population in 2 years

Trait	Chr	QTL	Position	LOD	Add	*R*^*2*^ (%)	Trait	Chr	QTL	Position	LOD	Add	*R*^*2*^ (%)
11RT							12RT	1	*qBR1–1*	RM297-RM486	2.98	0.54	3.95
	6	*qBR6*	L6ID3F-ZH6111	19.39	1.40	24.93		6	*qBR6*	L6ID3F-ZH6111	13.73	1.14	19.91
	7	*qBR7–1*	RM214-RM5543	7.83	0.89	8.87		7	*qBR7–1*	RM214-RM5543	3.18	0.55	4.12
	11	*qBR11–1*	RM229-RM457	5.17	1.08	6.57							
								11	*qBR11–2*	RM457-YH43	6.94	34.7	5.49
11RH							12RH	4	*qBR4–2*	RM241-RM317	3.44	0.49	2.51
	6	*qBR6*	ZH6111-RM20069	28.39	1.90	36.48		6	*qBR6*	L6ID3F-ZH6111	28.35	1.31	25.27
	7	*qBR7–1*	RM214-RM5543	14.98	1.36	16.47		7	*qBR7–1*	RM214-RM5543	29.33	1.43	26.31
								11	*qBR11–1*	RM229-RM457	3.2	0.61	2.42
11RN							12RN	1	*qBR1–2*	RM5-RM488	10.9	24.76	8.55
								2	*qBR2*	ZH282-RM71	2.59	44.12	8.92
								3	*qBR3*	RM3441-RM232	4.46	50.77	12.72
	4	*qBR4–1*	RM471-RM241	3.69	42.68	12.31		4	*qBR4–1*	RM471-RM241	7.64	40.01	18.47
	4	*qBR4–2*	RM241-RM317	2.53	45.94	8.82		4	*qBR4–2*	RM241-RM317	6.76	46.65	16.92
	6	*qBR6*	L6ID3F-ZH6111	33.94	50.47	31.91		6	*qBR6*	L6ID3F-ZH6111	29.23	42.24	28.95
	7	*qBR7–1*	RM214-RM5543	30.17	50.02	27.94		7	*qBR7–1*	RM214-RM5543	20.75	35.94	18.71
								7	*qBR7–2*	RM432-RM21691	4.61	17.06	3.43
	8	*qBR8*	RM404-RM210	7.80	38.08	18.89							
								12	*qBR12*	RM5927-RM6296	8.02	−33.18 ^a^	9.58

Note: Chr, Chromosome. LOD, logarithm of odds. Add, the additive effect of each QTL. *R*^*2*^, Goodness of fit, represent the phenotypic variance explained by each QTL. ^a^, the resistance effect of QTL come from Jin23B. 11RT and 12RT, leaf blast resistance at tillering stage in 2011 and 2012. 11RH and 12RH, leaf blast resistance at heading stage in 2011 and 2012. 11RN and 12RN, neck blast resistance at maturation stage in 2011 and 2012

For leaf blast resistance at tillering stage, five QTLs were detected on chromosome 1, 6, 7 and 11 (Table 3; Fig. 4). Among them, two QTLs, *qBR6* and *qBR7–1* were detected in both year, three QTLs, *qBR1–1*, *qBR11–1* and *qBR11–2* were detected only in 1 year (Table 3; Fig. 4). The QTL flanked by L6ID3F and ZH6111 on chromosome 6, *qBR6*, was detected in both year and explained 24.93% of the phenotypic variation in 2011 and 19.91% of the phenotypic variation in 2012. The QTL, *qBR7–1*, flanked by RM214 and RM5543 on chromosome 7 was detected in both years and accounted for 8.87% and 4.12% of the phenotypic variation, respectively. Whereas *qBR11–1* and *qBR11–2* were detected only in 1 year and accounted for 6.57% and 5.49% of the phenotypic variation.

For leaf blast resistance at heading stage, four QTLs were detected on chromosome 4, 6, 7 and 11 (Table 3; Fig. 4). Two QTLs, *qBR6* and *qBR7–1*, were detected in both year and the QTLs explained the phenotypic variance were 36.48% and 16.47% in 2011, respectively, and 25.27% and 26.31% in 2012, respectively. Two QTL, *qBR4–2* and *qBR11–1*, were located flanked by RM241-RM317 on chromosome 4, and RM229-RM547 on chromosome 11, respectively. The QTLs *qBR4–2* and *qBR11–1* were detected only in 2012 and accounted for 2.51% and 2.42% of the phenotypic variation, respectively.

For neck blast resistance at maturation stage, ten QTLs were detected on chromosome 1, 2, 3, 4, 6, 7 and 12 (Table 3; Fig. 4). Among them, four QTLs, *qBR4–1*, *qBR4–2*, *qBR6* and *qBR7–1* were detected in both year, and explained the phenotypic variance were 12.31%, 8.82%, 31.91% and 27.94%, respectively, in 2011, and were 18.47%, 16.92%, 28.95% and 18.71%, respectively, in 2012. Six QTLs, *qBR1–2*, *qBR2*, *qBR3*, *qBR7–2*, *qBR8* and *qBR12* were detected only in 1 year (Table 3; Fig. 4), and explained the phenotypic variance were 8.55%, 8.92%, 12.72%, 3.43%, 18.89% and 9.58%, respectively.

Among the thirteen QTLs in the Jin23B/QingGuAi3 population, the gene effect of twelve QTLs come from donor parent QingGuAi3, in contrast, the gene effect of *qBR12* come from Jin23B. The QTLs, *qBR12*, flanked by RM5927 and RM6296 on chromosome 12, was detected only in 2012 at maturation stage and explained phenotypic variations of 9.58% (Table 3). Three QTLs, *qBR4–2*, *qBR6* and *qBR7–1* were detected in both year at tillering and heading stages for leaf blast and maturation stage for neck blast; three QTL, *qBR1–1*, *qBR11–1* and *qBR11–2*, were detected for leaf blast at tillering or heading stages; five QTLs, *qBR1–2*, *qBR2*, *qBR3*, *qBR4–1* and *qBR7–2* were detected for neck blast at maturation stage.

### Validate the Genetic Effect of *qBR3–3* and *qBR6* from CR071 and *qBR6* and *qBR7–1* from QingGuAi3

**Fig. 5:**
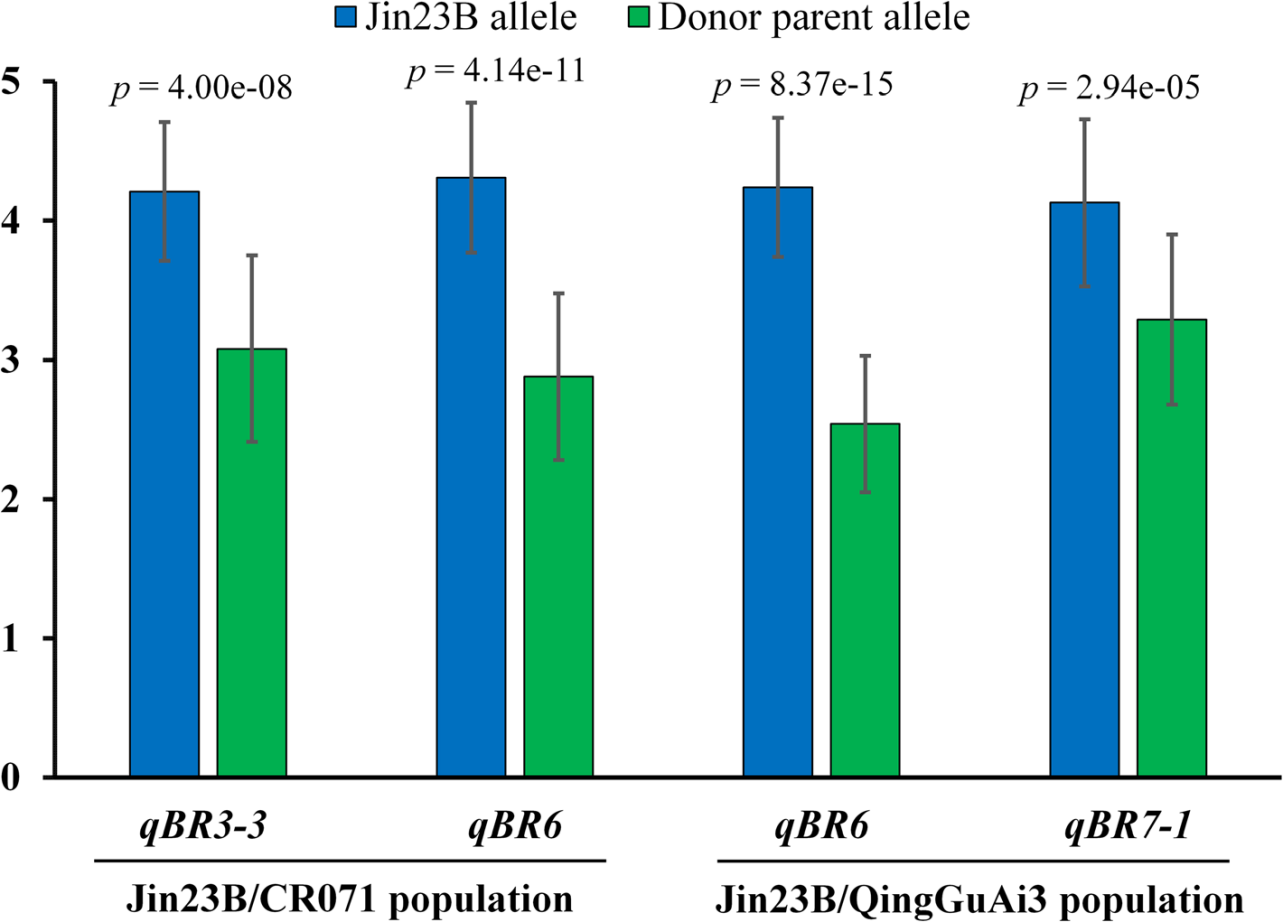
Genetic effects of *qBR3–3* and *qBR6* from CR071 and *qBR6* and *qBR7–1* from QingGuAi3. Blue bar represent alleles from Jin23B, Green bar represent alleles from donor parent. *P*-value based on two-way *t-test*. Error bars are based on standard deviation of each genotype

The BC_4_F_2_ segregation population of *qBR3–3* and *qBR6* from CR071 and *qBR6* and *qBR7–1* from QingGuAi3 were used to confirm the genetic effect of these QTLs. The *qBR3–3* and *qBR6* loci from CR071 increased blast resistance by 1.13 and 1.43, respectively, on leaf blast at tillering stage in 2013 (Fig. 5). The *qBR6* and *qBR7–1* loci from QingGuAi3 increased blast resistance by 1.70 and 0.83, respectively, on leaf blast at tillering stage in 2013 (Fig. 5).

## Discussion

QTL mapping using advanced backcross population was proposed as an effective molecular breeding technique for incorporating valuable genes from exotic sources into an adapted background (Eizenga et al. 2013; Tanksley and Nelson 1996). With this method, QTL from the donor parents are detected by backcrossing with the adapted parent to eliminate most of the unwanted genes from the donor parent. In addition, using advanced backcross population can accelerate the crop improvement process because near-isogenic lines containing the desired QTL (genes) from the donor in the background of the recurrent parent can be selected from the advanced backcross population. Several studies have been performed to identify QTLs in advanced backcrossing. For example, Thomson et al. (2003) mapped quantitative trait loci for yield, yield components and morphological traits in rice using a BC_2_F_1_ and BC_2_F_2_ population by composite interval mapping and Windows QTL Cartographer 1.21. And Eizenga et al. (2013) mapped sheath blight and blast quantitative trait loci in two different advanced backcross populations. In our study, we developed two advanced backcross population with the objective of introgression of useful resistance genes from CR071 and QingGuAi3 into Jin23B. Using this strategy, we mapped 16 blast resistance QTLs from the Jin23B/CR071 population and 13 blast resistance QTLs from the Jin23B/QingGuAi3 population. We also obtained maintainer lines resistant to blast in the background of Jin23B, which can be used for rice blast resistance breeding.

To date, over 100 blast resistant genes or QTLs have been identified (Su et al. 2015; Vasudevan et al. 2016; Xiao et al. 2017; Zheng et al. 2016). Among them, 37 genes have been cloned (Wang et al. 2017; Wang et al. 2019; Zhao et al. 2018). Many of these resistance genes are clustered on rice chromosomes 6, 11 and 12. Notably, at least 11 resistance genes—including *Pi2*, *Pi9*, *Piz*, *Pizt*, *Pigm*, *Pi22*, *Pi25*, *Pi26*, *Pi40*, *Pi42* and *Pi50*—are concentrated in the short-arm region near the centromere of chromosome 6. In this study, a QTL *qBR6* located between RM539 and R19951 in the Jin23B/CR071 population have a significant resistance to rice blast in both 2 years. When we further compared the position of this region to previous studies, we found that it contained *Pi2*, *Pi9*, *Pigm*, *Pizt* and *Pi50*, which are cloned blast resistance genes (Deng et al. 2017; Qu et al. 2006; Su et al. 2015; Zhou et al. 2006). QTL *qBR6* located between L6ID3F and ZH6111 in the Jin23B/QingGuAi3 population was also overlapped the *Pi2*/*Pi9* gene cluster on chromosome 6. Thus, *qBR6* identified in two population may be the allele of *Pi2*, *Pi9*, *Pigm*, *Pizt* or *Pi50*.

QTLs *qBR1* was located between RM237 and RM486 on chromosome 1 in the Jin23B/CR071 population, and the QTL *qBR1–2* was located between RM297 and RM486 in the Jin23B/QingGuAi3 population, in this region, blast resistance gene *Pi37*, *Pish* and *Pi35(t)* had previously been reported (Lin et al. 2007; Nguyen et al. 2006; Takahashi et al. 2010). The blast resistance gene *Pi37* and *Pish* have been cloned, which encode a NBS-LRR protein. *Pi35(t)* was identified in a QTL analysis of a population derived from the *Japonica* rice cultivar Hokkai 188 and the *Indica* rice cultivar Danghang-Shali. The resistance conferred by *Pi35(t)* to *M. oryzae* is classified as partial resistance (quantitative) rather than true resistance (qualitative). It is possible that *qBR1* from Jin23B/CR071 population and *qBR1–2* from Jin23B/QingGuAi3 population are allelic to either *Pi37*, *Pish* or *Pi35(t)*. QTLs *qBR2–3* located between RM530 and RM213 on chromosome 2 in the Jin23B/CR071 population, was close to the cloned gene *Pib* (Wang et al. 1999)*.* The neck blast resistance QTLs, *qBR11–1*, was located between RM21 and RM590 on chromosome 11 in the Jin23B/CR071 population, in this region, blast resistance gene *Pikm, Pik-h and Pik-p* had previously been reported (Ashikawa et al. 2008; Yuan et al. 2011; Zhai et al. 2014). It is possible that *qBR11–1* is allelic to either *Pikm, Pik-h* or *Pik-p*. In the Jin23B/QingGuAi3 population, QTL *qBR7–1* located between RM214 and RM5543 on chromosome 7 have a significant resistance to rice blast, in this region few gene have been reported, thus *qBR7–1* may be a new gene.

Rice cultivars with durable blast resistance have been recognized in several production systems. The durable resistance of these cultivars is associated with polygenic partial resistance that shows no evidence of race specificity. This partial resistance is expressed as fewer and smaller lesions on the leaf blade but latent period does not appear to be an important component. Many blast resistant varieties with single resistance genes lose resistance after a few years; that is, they have or had nondurable resistance (Babujee and Gnanamanickam 2000). Varieties with durable resistance may contain more than one resistance gene (Zhu et al. 2012). Many studies have been performed to pyramid resistances gene into rice varieties. Hittalmani et al. (2000) pyramided three blast resistance genes *Pi1*, *Piz-5* and *Pita* into rice variety CO39 and Jiang et al. (2012) pyramided three blast resistance genes *Pi1*, *Pi2* and *D12* into rice variety Jin23B. Their results confirmed that pyramiding of blast resistance genes is an effective way to develop highly resistant varieties. In our study, the donor parent CR071 and QingGuAi3 have a high level and durable resistance to blast over past decades, and have been used as donor parent for blast resistance in breeding in China. In the study, two advanced backcross population were constructed for analyses the genetic mechanism of blast resistance in CR071 and QingGuAi3, and major and minor blast resistance QTLs were identified in the donor parents. QTL effect analyses suggested that major and minor QTLs interaction is the genetic basis for durable blast resistance for CR071 and QingGuAi3 in the past decade in Wuling mountain area in China.

## Conclusions

Overall, the mapping results showed that sixteen blast resistance QTLs were identified in the Jin23B/CR071 backcross population, in which, QTLs *qBR1*, *qBR2–3* and *qBR8* were detected in both year for leaf blast and neck blast resistance; five QTLs *qBR2–1*, *qBR2–2*, *qBR3–3*, *qBR6* and *qBR12* were detected in both year for leaf blast at tillering and heading stages; eight QTLs *qBR3–1*, *qBR3–2*, *qBR4*, *qBR7–1*, *qBR7–2*, *qBR11–1*, *qBR11–2* and *qBR11–3* were detected for neck blast in maturation stage. Thirteen blast resistance QTLs were identified in Jin23B/QingGuAi3 population, in which, three QTLs, *qBR4–2*, *qBR6* and *qBR7–1* were detected in both year at tillering and heading stages for leaf blast and maturation stage for neck blast; three QTL, *qBR1–1*, *qBR11–1* and *qBR11–2*, were detected for leaf blast at tillering or heading stages; six QTLs, *qBR1–2*, *qBR2*, *qBR3*, *qBR4–1, qBR7–2* and *qBR12* were detected for neck blast at maturation stage.

## Additional Files

**Additional File 1.** Figure S1 Marker ratio of Jin23B homozygous genotype of each plant in two BC_3_F_1_ background population. a, marker ratio of Jin23B homozygous genotype of each plant in Jin23B/CR071 background population. b, marker ratio of Jin23B homozygous genotype of each plant in Jin23B/QingGuAi3 background population.

**Additional File 2.** Table S1 The correlation between six traits in Jin23B/CR071 background population

**Additional File 3.** Table S2 The correlation detection between six traits in Jin23B/QingGuAi3 background population

## Abbreviations

11RT: Leaf blast resistance at tillering stage in 2011
11RH: Leaf blast resistance at heading stage in 2011
11RN: Neck blast resistance at maturation stage in 2011. 12RT: Leaf blast resistance at tillering stage in 2012
12RH: Leaf blast resistance at heading stage in 2012
12RN: Neck blast resistance at maturation stage in 2012
CIM method: Composite interval mapping method
NBS-LRR gene: Nucleotide-binding site leucine-rich repeat gene
QTLs: Quantitative trait loci
R gene: Resistance gene
SSR: Simple sequence repeat
