# Supplementary figures and images for: Identification of Blast Resistance QTLs Based on Two Advanced Backcross Populations in Rice
Source: Rice (N Y). 2020 Jun 1;13:31. doi: 10.1186/s12284-020-00392-6 (PMC7266886; doi:10.1186/s12284-020-00392-6)

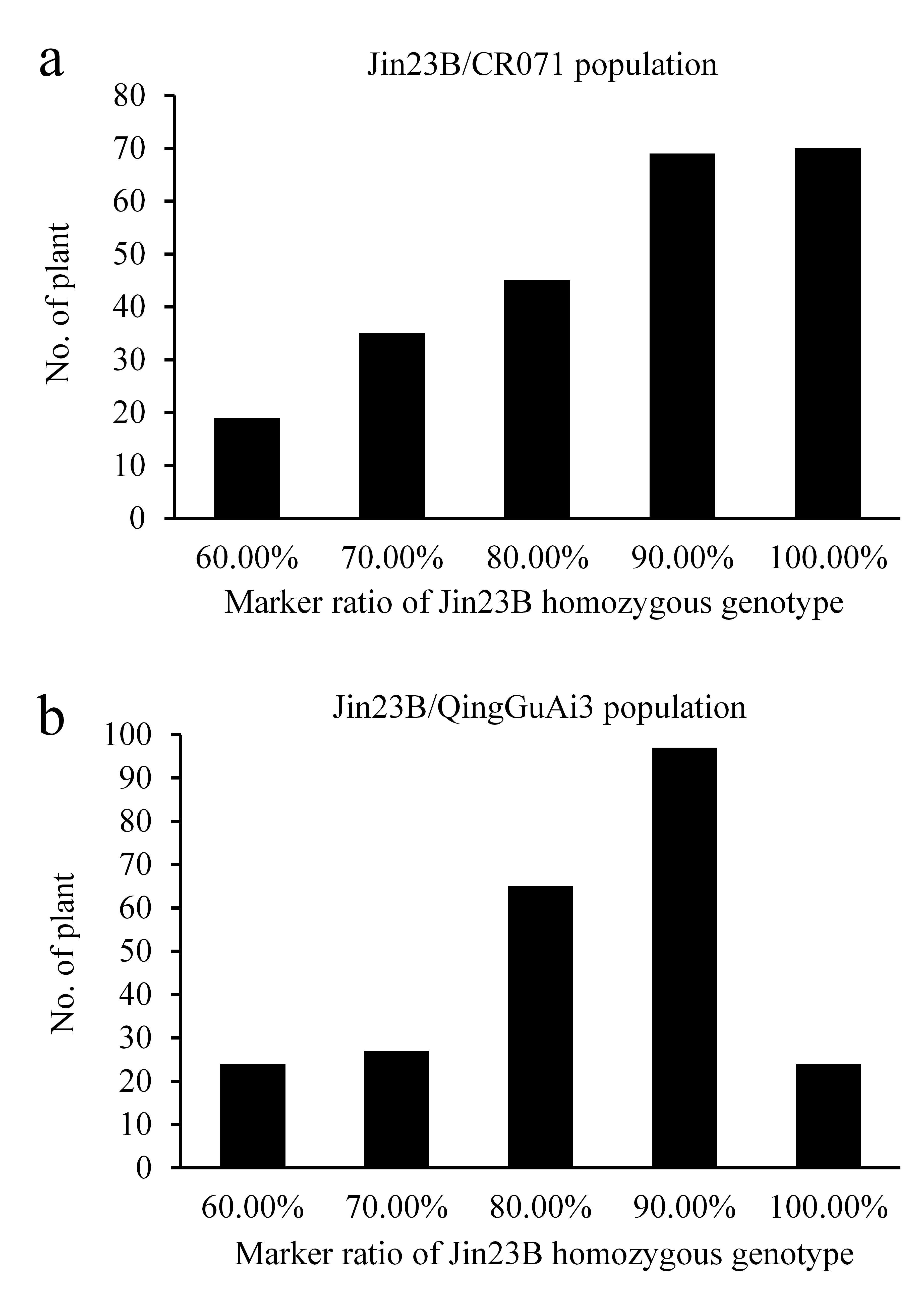

Supplement: Supplementary file 1 — Additional File 1. Figure S1 Marker ratio of Jin23B homozygous genotype of each plant in two BC3F1 background population. a, marker ratio of Jin23B homozygous genotype of each plant in Jin23B/CR071 background population. b, marker ratio of Jin23B homozygous genotype of each plant in Jin23B/QingGuAi3 background population. [file 12284_2020_392_MOESM1_ESM.tif]
