# Supplementary material for: Identification of Blast Resistance QTLs Based on Two Advanced Backcross Populations in Rice
Source: Rice (N Y). 2020 Jun 1;13:31. doi: 10.1186/s12284-020-00392-6 (PMC7266886; doi:10.1186/s12284-020-00392-6)
Supplement: Supplementary file 2 — Additional File 2. Table S1 The correlation between six traits in Jin23B/CR071 background population [file 12284_2020_392_MOESM2_ESM.docx]

Table S1 The correlation between six traits in Jin23B/CR071 background population

|  | 11RT | 11RH | 11RN | 12RT | 12RH |
| --- | --- | --- | --- | --- | --- |
| 11RH | 0.744^**^ |  |  |  |  |
| 11RN | 0.590^**^ | 0.646^**^ |  |  |  |
| 12RT | 0.931^**^ | 0.911^**^ | 0.655^**^ |  |  |
| 12RH | 0.870^**^ | 0.903^**^ | 0.633^**^ | 0.952^**^ |  |
| 12RN | 0.625^**^ | 0.676^**^ | 0.898^**^ | 0.698^**^ | 0.665^**^ |

Note: 11RT and 12RT, leaf blast resistance at tillering stage in 2011 and 2012. 11RH and 12RH, leaf blast resistance at heading stage in 2011 and 2012. 11RN and 12RN, neck blast resistance at maturation stage in 2011 and 2012. ^**^, Significant at the 0.01 level (bilateral).
